# Assessment of Financial Relationships Between Otorhinolaryngologists and Pharmaceutical Companies in Japan Between 2016 and 2019

**DOI:** 10.7759/cureus.43633

**Published:** 2023-08-17

**Authors:** Sae Kamamoto, Akihiko Ozaki, Anju Murayama

**Affiliations:** 1 School of Medicine, Hamamatsu University, Hamamatsu, JPN; 2 Surgery, Teikyo University Graduate School of Public Health, Tokyo, JPN; 3 School of Medicine, Tohoku University, Sendai, JPN

**Keywords:** medical ethics, public health policy, health policy and economics, financial conflicts of interest, ethics, ethics and professionalism, otolaryngologist, industry payment, japan, conflict of interest

## Abstract

Introduction: There are prevalent financial relationships between physicians and the pharmaceutical industry in medical specialties, including otorhinolaryngology. Although these relationships might cause conflicts of interest, no studies have assessed the size and contents of the financial relationships between otorhinolaryngologists and pharmaceutical companies in Japan. This study aims to evaluate the magnitude, prevalence, and trend of the financial relationship between Japanese otolaryngologists and pharmaceutical companies.

Methods: Using payment data publicly disclosed by 92 pharmaceutical companies, we examined the size, prevalence, and trend in personal payments made to the otorhinolaryngologist board certified by the Japanese Society of Otorhinolaryngology-Head and Neck Surgery (JSO-HNS) between 2016 and 2019 in Japan. Furthermore, differences in payments were evaluated by whether otolaryngologists were clinical practice guideline authors, society board members, and academic journal editors or not. Trends in payments were evaluated by generalized estimating equations.

Results: Of 8,190 otorhinolaryngologists, 3,667 (44.8%) were paid a total of $13,873,562, in payments for lecturing, consulting, and writing by 72 pharmaceutical companies between 2016 and 2019. The median four-year combined payment per physician was $1,022 (interquartile range: $473-$2,526). Top 1%, 5%, and 10% of otorhinolaryngologists received 42.3% (95% confidence interval (95% CI): 37.2%-47.4%), 69.3% (95% CI: 65.9%-72.8%), and 80.6% (95% CI: 78.3%-82.9%) of overall payments, respectively. The median payments per physician were significantly higher among otorhinolaryngologists authoring clinical practice guidelines ($11,522), society board members ($22,261), and journal editors ($35,143) than those without. The payments and number of otorhinolaryngologists receiving payments remained stable between 2016 and 2019.

Conclusion: This study demonstrates that a minority but a large number of otorhinolaryngologists received personal payments from pharmaceutical companies for the reimbursement of lecturing, consulting, and writing in Japan. Large amounts of these personal payments were significantly concentrated on a small number of leading otorhinolaryngologists.

## Introduction

Although collaborations between industry and healthcare professionals can bring breakthroughs in medicine, several medical scandals and limited transparency in the financial relationships between healthcare professionals and pharmaceutical companies led to concern for the undue influence of financial relationships on patient care. Since 2013, the Japan Pharmaceutical Manufacturers Association (JPMA), the largest pharmaceutical trade organization in Japan, has required all pharmaceutical companies belonging to the JPMA, whose share account for more than 80% of total sales for pharmaceutical products in Japan [1], to disclose their payments made to healthcare professionals for lecturing, consulting, and writing, based on the JPMA voluntary transparency guidance [2,3]. This voluntary payment disclosure by pharmaceutical companies has enabled the evaluation of the detailed magnitude of the financial relationships between healthcare professionals and pharmaceutical companies in several specialties [4-12].

As shown in previous studies in the United States, there are large and prevalent financial transfers from pharmaceutical industries to otorhinolaryngologists for various purposes [13-17], as well as other specialty physicians [18-25]. The payments from pharmaceutical companies often disproportionately concentrate on small numbers of physicians in leading and authoritative positions who are required to be independent and unbiased from any industries [4,26-30], namely, key opinion leaders [31,32]. This trend would exist among Japanese otorhinolaryngologists, considering previous studies showing that there were substantial and prevalent financial relationships between leading physicians and pharmaceutical companies in other specialties in Japan [7,9,33-35]. However, there was a lack of assessment regarding the whole picture of the financial relationships between pharmaceutical companies and otorhinolaryngologists in Japan. Thus, this study aimed to evaluate the magnitude, prevalence, and trend in personal payments made to otorhinolaryngologists by pharmaceutical companies for the latest years in Japan.

## Materials and methods

Study design and study participants

This retrospective study examined the magnitude and trends in financial relationships between pharmaceutical companies and all otorhinolaryngologists board-certified by the Japanese Society of Otorhinolaryngology-Head and Neck Surgery (JSO-HNS). As the JSO-HNS did not disclose the name list of board-certified otorhinolaryngologists for the previous years, we considered all board-certified otorhinolaryngologists in 2021. The JSO-HNS, established in 1893, is the sole and most authoritative professional medical society certifying otorhinolaryngologists in the field of otorhinolaryngology and head and neck surgery in Japan. The JSO-HNS has contributed to training otorhinolaryngologists, funded clinical trials and basic research, published many clinical practice guidelines for otorhinolaryngological diseases, and issued the English-language academic journal (*Auris Nasus Larynx*). This study defined leading otorhinolaryngologists as board-certified otorhinolaryngologists authoring clinical practice guidelines, board members of the JSO-HNS, and editorial members of *Auris Nasus Larynx*.

Data collection

As the JSO-HNS did not disclose the name list of board-certified otorhinolaryngologists for the previous years, the name, practicing region, and prefecture of all board-certified otorhinolaryngologists in 2021 were extracted from the official webpage of the JSO-HNS. Furthermore, we collected the name of all clinical practice guideline authors issued and reviewed by the JSO-HNS between 2015 and 2020 (including one year before and after the payment period), the JSO-HNS board members in 2018-2019 and 2020-2021, and editorial members of *Auris Nasus Larynx* in April 2022. For the data collection of society board members, we previously collected the name list of the JSO-HNS in 2018-2019 and 2020-2021 [29]. As the *Auris Nasus Larynx* did not publicly provide the name list of editorial board members in previous years, we collected the latest editorial members of* Auris Nasus Larynx* in April 2022.

The payments concerning lecturing, consulting, and writing paid to the board-certified otorhinolaryngologists were extracted from a total of 92 pharmaceutical companies belonging to the JPMA between 2016 and 2019. The period of payment data collection was determined by our availability of data collection. The companies have published and updated the payment data each year on their company websites. The payment data for all companies belonging to the JPMA were collected from 2016 and May 2022, and the payment data from 2019 are the latest analyzable data in Japan. Payment categories were described in our previous study and the JPMA transparency guideline [3,35]. The detailed procedure of the payment collection was noted previously [4].

Analysis

First, the payment data were descriptively analyzed. Payments per physician were also calculated only for physicians receiving payments each year, as in other previous studies [9,16,18,36-38]. Second, the payment concentration was evaluated by the shares of the payment values held by the top 1%, 5%, 10%, and 25% of the otorhinolaryngologists and the Gini coefficient at the physician level. The Gini index ranges from 0 to 1, and the greater the Gini index, the greater the disparity in the distribution of payments [10,34,39-41]. Third, we calculated descriptive statistics and evaluated payment differences among the leading otorhinolaryngologists, including guideline authors, society board members, journal editors, and other otorhinolaryngologists. The differences in payments by each variable were evaluated by chi-square and Fisher's exact tests for the proportion of otorhinolaryngologists receiving payments and by Mann-Whitney U test for payment values per otorhinolaryngologist.

Furthermore, the linear log-linked Poisson regression model was used to assess the association between the relative risk of payment receipt and the otorhinolaryngologist characteristics. To account for the highly skewed distribution of payment values, a negative binomial regression model was employed to evaluate the association between the relative monetary value of payments per physician and the otorhinolaryngologist characteristics, including practicing prefecture, participation in clinical guideline development, JSO-HNS board membership, and editorial membership of the society journal, as in previous studies [9,35,37,38]. Finally, we evaluated the trends in payments per physician and the number of physicians receiving payments between 2016 and 2019 by the population-averaged generalized estimating equation (GEE) with the panel data of the annual payments. As the payment distribution was highly skewed, the negative binomial GEE model for the payment values per physician and linear log-linked GEE model with Poisson distribution for the number of otorhinolaryngologists with payments were selected [10,12,42]. The payment values were converted from Japanese yen (¥) to US dollars ($) using the 2019 average monthly exchange rates of ¥109.0 per $1. All analyses were conducted using Microsoft Excel, version 16.0 (Microsoft Corporation, United States) and Stata statistical software, version 15 (StataCorp, 2017, College Station, TX: StataCorp LLC.).

Ethical approval

The Ethics Committee of the Medical Governance Research Institute approved this study (approval number: MG2018-04-20200605; approval date: June 5, 2020). As this retrospective analysis only included publicly available information, informed consent was waived by the ethics committee.

Patient and public involvement

No patient was involved in this study.

## Results

Overall and per-otorhinolaryngologist payments

At the time of this study, we identified 8,190 otorhinolaryngologists board-certified by the JSO-HNS. Of the 8,190 otorhinolaryngologists, 3,667 (44.8%) were paid a total of $13,873,562, entailing 22,076 contracts in payments for lecturing, consulting, and writing by 72 pharmaceutical companies between 2016 and 2019 (Table 1). The median payment per physician was $0 (interquartile range (IQR): $0-$851) for overall otorhinolaryngologists. For otorhinolaryngologists receiving payments, the median payment per physician was $1,022 (IQR: $473-$2,526), while the average payment was $3,783 (standard deviation (SD): $14,349). The median payment contracts and number of companies making payments per physician were 3.0 (IQR: 1.0-6.0) and 2.0 (IQR: 1.0-4.0) over the four years, respectively. One otorhinolaryngologist received a maximum payment of $490,081 and 332 payment contracts.

**Table 1:** Summary of personal payments from pharmaceutical companies to board-certified otorhinolaryngologists between 2016 and 2019 SD: standard deviation, IQR: interquartile range

Variables	
Total	
Payment values, $	13,873,562
Instances, No.	22,076
Companies, No.	72
Average per physician (SD)	
Payment values, $	3,783 (14,349)
Instances, No.	6.0 (13.6)
Companies, No.	3.0 (3.0)
Median per physician (IQR)	
Payment values, $	1,022 (473‒2,526)
Instances, No.	3.0 (1.0‒6.0)
Companies, No.	2.0 (1.0‒4.0)
Range	
Payment values, $	28‒490,081
Instances, No.	1.0‒332
Companies, No.	1.0‒27.0
Category of payments	
Lecturing	
Payment value, $ (%)	11,968,045 (84.8)
Instances, No. (%)	18,714 (84.8)
Physicians, No. (%)	3373 (41.2)
Consulting	
Payment value, $ (%)	1,075,487 (7.8)
Instances, No. (%)	2,121 (9.6)
Physicians, No. (%)	1112 (13.6)
Writing	
Payment value, $ (%)	701,495 (5.1)
Instances, No. (%)	1,075 (4.9)
Physicians, No. (%)	494 (6.0)
Other	
Payment value, $ (%)	128,534 (0.9)
Instances, No. (%)	168 (0.8)
Physicians, No. (%)	113 (1.4)

Payments by category and payment concentration

Payments for lecturing occupied 86.3% of overall monetary values ($11,968,045) and 84.8% of overall payment contracts (18,714 contracts) between 2016 and 2019. Of 8,190 eligible otorhinolaryngologists, 3,373 (41.2%), 1,112 (13.6%), and 494 (6.0%) received one or more compensation payments for lecturing, consulting, and writing from the pharmaceutical companies over the four years, respectively.

While majority of otorhinolaryngologists did not receive any payments from the pharmaceutical companies over the four years, top 1%, 5%, 10%, and 25% of otorhinolaryngologists received 42.3% (95% CI: 37.2%-47.4%), 69.3% (95% CI: 65.9%-72.8%), 80.6% (95% CI: 78.3%-82.9%), and 94.8% (95% CI: 94.1%-95.5%) of overall payments, respectively. The Gini coefficient for the four-year combined payments per physician was 0.889, indicating that the payments disproportionately concentrated on small numbers of otorhinolaryngologists.

Payments to leading otorhinolaryngologists: clinical practice guideline authors, society board members, and academic journal editors

We identified a total of 139 individual authors from eight clinical practice guidelines accredited or authorized by the JHO-HNS between 2015 and 2020 (Table 2). Of the 139 authors, 101 (72.7%) authors were board-certified otorhinolaryngologists, and 94 (93.1%) received one or more personal payments for lecturing, consulting, and writing compensations. A total of $2,435,239 (17.6% ($2,435,239 out of $13,873,562) of the overall personal payments from the companies) were made to 94 otorhinolaryngologists authoring clinical practice guidelines. The aggregated payment per physician was significantly higher among otorhinolaryngologists authoring clinical practice guidelines than that of otorhinolaryngologists not involved in authoring guidelines ($11,522 (IQR: $3,090-$32,390) vs. $0 (IQR: $0-$817), p<0.001).

**Table 2:** Payments to the board-certified otorhinolaryngologists with leading roles between 2016 and 2019 a. The difference in proportion of otorhinolaryngologists with payments was evaluated by the chi-square test and fisher exact test. b. The difference in payments per otorhinolaryngologist was evaluated by the Mann-Whitney U test for the two groups. c. The interaction between continuous variable society board membership and journal editorial membership were included in multivariable regression models. The relative risk for the interaction was 0.81 (95% CI: 0.63–1.03) and relative monetary value for the interaction was 2.29 (95% CI: 0.63–8.38). SD: standard deviation, IQR: interquartile range, 95% CI: 95% confidence interval

	Physician with payments		Payment per physician $			Relative payments			
	Number (%)	P value^a^	Average (SD)	Median (IQR)	P value^b^	Relative risk for receiving payments (95% CI)	P value	Relative monetary value (95% CI)	P value
Clinical practice guideline									
Non-guideline author otorhinolaryngologists	3,573 (44.2)	<0.001	1,414 (8,751)	0 (0-817)	<0.001	Ref.	<0.001	Ref.	<0.001
Otorhinolaryngologists authoring guideline	94 (93.1)		24,111 (33,621)	11,522 (3,090-32,390)		1.96 (1.82-2.12)		13.03 (9.55-17.79)	
Board membership^c^									
Non-board members	3633 (44.6)	<0.001	1,550 (9,109)	0 (0-831)	<0.001	Ref.	<0.001	Ref.	<0.001
Board membership	34 (94.4)		34,298 (44,388)	22,261 (4,537-50,331)		1.47 (1.20-1.79)		8.57 (3.04-24.17)	
Journal editorial membership^c^									
Non-editor otorhinolaryngologists	3,648 (44.7)	<0.001	1,603 (9,365)	0 (0-851)	<0.001	Ref.	<0.001	Ref.	0.001
Editor otorhinolaryngologists	19 (100)		40,746 (46,059)	35,143 (7,733-50,373)		1.21 (1.11-1.33)		0.54 (0.38-0.77)	

All 36 board members of the JSO-HNS during the 2018-2019 and 2020-2021 periods were board-certified otorhinolaryngologists. Of the 36 board-certified otorhinolaryngologists with the JSO-HNS board membership, 34 (94.4%) received a total of $1,234,715 (8.9% of overall payments) and a median payment of $22,261 (IQR: $4,537-$50,331) per physician (Table 2). Both the proportion of otorhinolaryngologists receiving payments (94.4% vs. 44.6%, p<0.001) and the payments per physician ($22,261 (IQR: $4,537-$50,331) vs. $0 (IQR: $0-$831), p<0.001) were significantly higher for the otorhinolaryngologists positioned as the JSO-HNS board member than those without board membership.

There were 20 Japanese editors of *Auris Nasus Larynx,* and among them, 19 editors were board-certified otorhinolaryngologists. All 19 (100%) board-certified otorhinolaryngologists who are editors of *Auris Nasus Larynx* received payments of $774,171 (5.6% of the overall payments) in total and $35,143 (IQR: $7,733-$50,373) in median per-physician payments from pharmaceutical companies (Table 2).

The multivariable Poisson regression model showed that clinical practice guideline authorship, JSO-HNS board membership, and editorial membership in the academic journal were significantly associated with 1.96 (95% CI: 1.82-2.12) times, 1.47 (95% CI: 1.10-1.79) times, and 1.21 (95% CI: 1.11-1.33) times higher likelihood to accept personal payments from pharmaceutical companies than those without authorships or memberships (Table 2). The multivariable negative binomial regression model indicated that clinical practice guideline authorship and JSO-HNS board membership were positively associated 13.04 (95% CI: 9.55-17.79) times and 8.57 (95% CI: 3.04-24.17) times higher monetary values in personal payments, while editorial membership in the academic journal was negatively associated with payment values.

The JSO-HNS required clinical practice guideline authors to declare their financial conflicts of interest (FCOIs) with the industry, and the authors disclosed their FCOIs in the guidelines. Meanwhile, there was no FCOI disclosure among the JSO-HNS board members and the academic journal editors.

Trends in personal payments between 2016 and 2019

The total annual payments from the pharmaceutical companies ranged from $3,356,647 in 2016 to $3,615,634 in 2017. A total of 1,988 (24.3%) otorhinolaryngologists in 2019 to 2,129 (26.0%) otorhinolaryngologists in 2018 received more than one personal payment from the companies in a single year (Table 3). The median annual payments per physician was $511 (IQR: $307‒$1,188) in 2016 to $619 (IQR: $473‒$1,328) in 2019, while the average annual payment per physician was $1,663 (SD: $5,505) to $1,761 (SD: $5,518). There were no significant annual changes in the total payments, payments per physician, and the number of otorhinolaryngologists receiving payments. A sensitivity analysis, limiting payments from 63 companies whose payment data were available throughout the four years, also confirmed that there were no significant annual changes in total payments, payment per physician, and the number of otorhinolaryngologists with payments between 2016 and 2019.

**Table 3:** Trend of personal payments from pharmaceutical companies to board-certified otorhinolaryngologists between 2016 and 2019 a. There were nine companies without payment data through the four years and were excluded from the trend analysis. SD: standard deviation, IQR: interquartile range

Variables	Payment year				Average yearly change (95%CI), %	P value
	2016	2017	2018	2019		
All pharmaceutical companies						
Total payments, $	3,356,647	3,615,634	3,463,336	3,437,945	-0.26 (-2.06‒2.59)	0.84
Average payments per physician (SD), $	1,663 (5,505)	1,761 (5,518)	1,627 (4,319)	1,729 (4,249)	0.27 (-2.72‒3.35)	0.86
Median payments per physician (IQR), $	511 (307‒1,188)	511 (307‒1,209)	613 (411‒1,211)	619 (473‒1,328)		
Range of payments per physician, $	28‒164,556	94‒151,906	92‒91,580	92­‒82,038		
Physicians with payments, n (%)	2,019	2,053	2,129	1,988	-0.083 (-1.34‒1.19)	0.90
Gini index	0.923	0.922	0.910	0.913	‒	‒
Pharmaceutical companies with 4-years payment data^a^						
Total payments , $	3,315,057	3,608,993	3,417,689	3,358,464	-0.18 (-3.06‒2.70)	0.90
Average payments per physician (SD), $	1,653 (5,491)	1,758 (5,509)	1,616 (4,290)	1,714 (4,219)	-0.18 (-3.14‒2.87)	0.91
Median payments per physician (IQR), $	511 (307‒1,188)	511 (307‒1,209)	613 (409‒1,211)	613 (473‒1,306)		
Range of payments per physician, $	28‒163,610	94‒151,906	92‒90,161	92‒82,038		
Physicians with payments, n (%)	2,005	2,053	2,115	1,959	-0.37 (-1.63‒0.90)	0.56
Gini index	0.923	0.922	0.911	0.914	‒	‒

Payments by company

**Figure 1:**
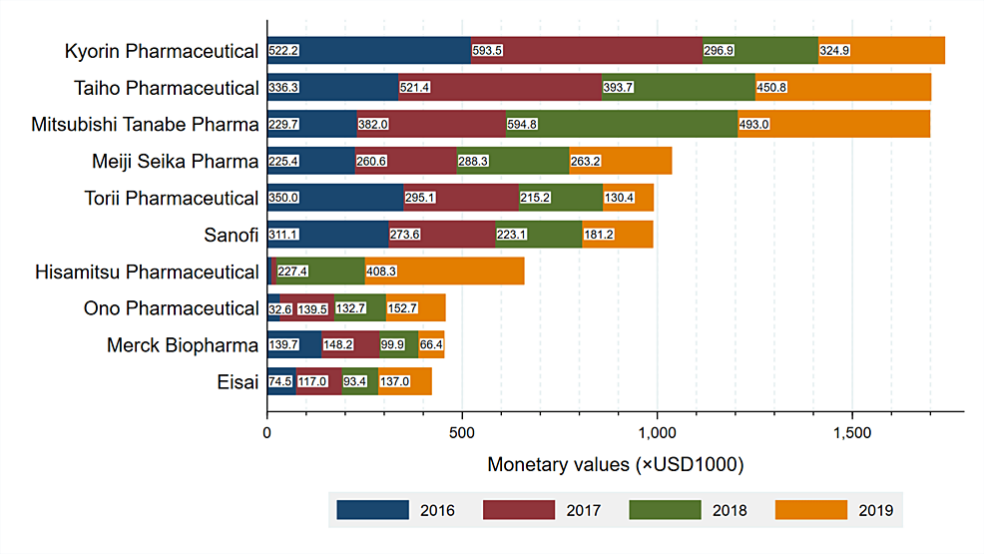
Payment trends by company Total payments made to all board-certified otorhinolaryngologists for lecturing, consulting, and writing between 2016 and 2019 by each company.

Total payments by company are described in Figure 1. Kyorin Pharmaceutical paid the largest personal payments to the board-certified otorhinolaryngologists, accounting for 12.6% ($1,745,682 out of $13,873,561) of overall payments. Similarly, payments from Taiho Pharmaceutical and Mitsubishi Tanabe Pharma, the second and third largest paying companies, accounted for 12.3% ($1,705,181) and 12.3% ($1,704,126) of overall payments, respectively. The payments from the top 10 companies occupied 73.3% of overall personal payments between 2016 and 2019. Most companies made personal payments for the purpose of lecturing to the board-certified otorhinolaryngologists.

## Discussion

This study demonstrates that a minority but a large number of otorhinolaryngologists received personal payments from pharmaceutical companies for the reimbursement of lecturing, consulting, and writing in Japan. Large amounts of these personal payments were significantly concentrated on a small number of otorhinolaryngologists with leading positions, such as clinical practice guideline authors, society board members, and academic journal editors in the field of otorhinolaryngology. We observed that the personal financial relationships between the otorhinolaryngologists and pharmaceutical companies had remained stable over the four years in Japan. Our findings show significant similarities and differences compared to previous studies assessing this issue in Japan and other developed countries.

First, this large sample-sized longitudinal observational study elucidated that 44.8% of all board-certified otorhinolaryngologists received a median personal payment of $1,022 from pharmaceutical companies. Previous studies in Japan reported that there was an increasing trend in physicians receiving payments from pharmaceutical companies in terms of the number of physicians with payments and payments per physician [5,10,35,42,43]. The proportion of physicians with payments and median four-year personal payments was from 62.0% in pulmonology [12] to 70.6% in medical oncology [5] and $2,210 in pulmonology [12] to $3,183 in infectious diseases [43], respectively. Smaller payments made to otorhinolaryngologists as observed in this study were consistent with many previous studies in the United States [13-15,18,44]. Pathak et al. found that US otorhinolaryngologists received the second lowest personal payments in surgical specialties between 2014 and 2015 [15]. Cvetanovich et al. [44] and Rathi et al. [13] reported that the trend of lowest payments made to the otorhinolaryngologists persisted since the launch of the Open Payments Program in 2013. Fewer expensive and novel drugs and the large number of otorhinolaryngologists could contribute to the lower payment values both in Japan and the US.

Second, the personal financial relationships between the otorhinolaryngologists and pharmaceutical companies remained stable over the four years at both low monetary payment values and number of otorhinolaryngologists with payments. In contrast to our findings, Morse et al. previously observed that there was an increasing trend in personal payments among the US otorhinolaryngologists between 2014 and 2016 [14], while the increasing trend was not observed in 2017 [16]. Meanwhile, even lower personal payments to otorhinolaryngologists significantly influence otorhinolaryngologists’ clinical practice, such as increasing brand-name prescriptions [45], prescribing more brand-name intranasal corticosteroids over generic alternatives [46], and performing more controversial treatment, such as sinus balloon catheter dilation [47,48]. Accumulating evidence strongly suggests that personal payments made by pharmaceutical companies significantly distort physicians’ prescribing patterns, which were potentially harmful to patients [45,47,49-56], increase healthcare costs [49,56-59], and lower patients’ trust in physicians [60-62], while many physicians have denied the influence and justified their personal financial relationships with industries [63-65].

In addition, our study directly demonstrated that large amounts of personal payments significantly concentrated on only a small number of otorhinolaryngologists positioned in authoritative and public roles, such as clinical practice guideline authors, society board members, and academic journal editors. A high concentration of payments on leading physicians, namely, key opinion leaders, is pervading in medicine worldwide [7,9,26-28,30,32,37,66-69]. Moynihan et al. elucidated that 72% of board members of 10 US professional medical societies in the highest financial burden disease areas accepted a median of $6,026 in personal payments from pharmaceutical and medical device companies between 2013 and 2018 [27]. Similarly, Saito et al. reported that 86.9% of the board members from 19 major Japanese professional medical societies received a median per-physician payment of $7,486 in 2016 [29]. Moreover, there are prevalent and large FCOIs among clinical practice guideline authors and journal editors in many developed countries [70-77]. Furthermore, many of the financial relationships between leading physicians and pharmaceutical companies are undisclosed to the public and underreported [4,11,27,33,34,43,75,78-84], as we found that the JSO-HNS did not disclose FCOIs among the board members and academic journal editors. Unlike leading physicians conducting clinical trials and research sponsored by the industry, leading physicians, such as clinical practice guideline authors, society board members, and academic journal editors, are necessary to manage and, if possible, be free from financial interest with the industry, as their financial interest with industry conflict with their primary interest [6,7,9-11,33,41,42,81,82,85-88]. Currently, FCOIs among clinical practice guideline authors are strictly managed by many guideline-developing organizations: the minority of guideline authors with FCOIs involve in guideline development, all FCOIs for the past three years are declared and disclosed by guideline authors, and the guideline chairperson is required to be free from any FCOIs with industry [11,81,84,86,88-90]. Several academic journals, such as the *Annals of Emergency Medicine*, the official journal of the American College of Emergency Physicians, and the *Journal of Urology*, the official journal of the American Urological Association, disclose the editors’ FCOIs on journal webpages [75]. Transparency and rigorous management are necessary for financial relationships between pharmaceutical companies and leading otorhinolaryngologists with authoritative and public positions.

Limitations

This study included several limitations: First, there would be underestimated payments made by non-member companies of the JPMA to the otorhinolaryngologists. However, as the member companies accounted for 80.8% of the total pharmaceutical sales in Japan in 2018 [1], such an underestimation of payments could be minimized by including data from all member companies. Second, the pharmaceutical companies were not required to disclose other categories of payments, such as meals, beverages, travel, and stock ownership, at an individual level, according to the JPMA guidance [3]. This could have led to underestimations of the extent and prevalence of overall financial relationships between otorhinolaryngologists and industries. Third, this study included otorhinolaryngologists as of November 2021, as the JSO-HNS did not disclose the name list of otorhinolaryngologists for previous years. Therefore, this study would have included otorhinolaryngologists who were not certified during the study period. Fourth, the payment magnitude and trend may not be applicable to other countries.

## Conclusions

Although a minority of otorhinolaryngologists board-certified by the JSO-HNS stably received personal payments from pharmaceutical companies for the reimbursement of lecturing, consulting, and writing between 2016 and 2019, large amounts of payments significantly concentrated on a relatively small number of otorhinolaryngologists. Leading otorhinolaryngologists, such as clinical practice guideline authors, society board members, and academic journal editors, significantly accepted far larger personal payments than those who were not.
